# Needlestick and Sharps Injuries Among Nursing Students in Palestine: Prevalence, Reporting Practices, and Associated Factors

**DOI:** 10.1155/nrp/4472112

**Published:** 2026-09-15

**Authors:** Fuad Farajalla, Nesreen Alqaissi, Mohammad Qtait, Yousef M. Jaradat, Areej Ashraf, Tasneem Ghannam, Bagdad Ajwah, Amal Najada, Sujood Dawadeh

**Affiliations:** ^1^ Nursing Department, Palestine Polytechnic University, Hebron, State of Palestine, ppu.edu

**Keywords:** needlestick injuries, nursing students, occupational exposure, patient safety, psychological stress, sharps injuries

## Abstract

**Background:**

Needlestick and sharps injuries (NSIs) are a major occupational hazard among nursing students. Although previous studies in Palestine have reported prevalence and awareness, analytically driven evidence identifying factors independently associated with NSIs remains limited.

**Aim:**

This study aimed to assess the prevalence, experiences, and reporting practices of NSIs and to identify factors independently associated with NSIs among nursing students in the southern West Bank, Palestine.

**Methods:**

A cross‐sectional study was conducted using convenience sampling among 398 bachelor’s nursing students from four universities. Data were collected using a structured questionnaire and analyzed using descriptive statistics, bivariate analyses, and multivariable logistic regression (*p* < 0.05).

**Results:**

The prevalence of NSIs was 28.6% (*n* = 114). Blood drawing (38.5%) and needle recapping (30.7%) were the most common causes. Most students reported incidents (83.3%), although fear of blame and lack of procedural knowledge persisted. In the multivariable analysis, higher perceived stress when handling sharps was significantly associated with increased odds of NSIs (aOR = 2.13; 95% CI: 1.35–3.37). Academic year, glove use, recapping behavior, and equipment availability were not significantly associated with NSIs.

**Conclusion:**

NSIs are relatively common among nursing students in this setting. The findings suggest that perceived stress is associated with NSI occurrence; however, due to the cross‐sectional design, causal inferences cannot be made. Further longitudinal research is needed to understand causal pathways, explore additional contributing factors, and inform effective prevention strategies.

## 1. Introduction

Needlestick and sharp injury (NSI) events are considered to pose significant occupational risk in healthcare facilities around the globe. NSI can be described as any skin puncture injury caused by hypodermic needles or other sharps contaminated with blood or other fluid from the human body or tissues [1, 2]. The vast knowledge about NSIs and the adoption of safety measures have not reduced their occurrence, indicating a possible knowledge–practice gap in these facilities [2]. One of the most alarming facts about NSI injury events in these facilities is the under‐reporting tendency, whereby it has been found that half, or rather more, of such incidents go unreported [3, 4].

Currently, a recent study suggests the occurrence of NSI at an estimated 43% incidence rate, synthesized from 113 studies, including in excess of 520,000 healthcare workers from 2000 through 2020 [5]. Researchers estimate that percutaneous exposure is linked to 40% to 65% of HBV and HCV infections in healthcare workers in developing regions [6]. This alarming statistic underscores the need for renewed efforts to mitigate these risks.

Nursing students are one of the most affected groups of healthcare professionals experiencing NSIs at teaching hospitals [7]. Due to less experience, higher workloads, and frequent use of needles and sharp objects while receiving clinical training, nursing students face a greater risk of an NSI [8]. A significant percentage of nursing students (19.1%) lack hepatitis B immunization, further increasing their risk [9].

There are many factors that can be responsible for the occurrence of NSIs. This includes the way in which nursing students are trained in their clinical skills and the type of training that is provided for them, including clinical competence, a high volume of patients, and the level of stress [10, 11]. Recent evidence also highlights the important role of emotional intelligence in nurses’ ability to manage occupational stress and safety‐related behaviors [12]. In addition, other factors related to an organization’s policies or a nurse’s skills also contribute to the increased vulnerability of nursing students to an NSI [13].

This study is conceptually informed by established occupational safety models that view adverse events as the result of interactions between multiple system levels rather than isolated individual errors. In particular, the Swiss Cheese Model conceptualizes injuries as occurring when latent system failures (e.g., inadequate supervision, resource limitations) align with active human factors (e.g., stress, unsafe practices) [14]. In parallel, human factors and occupational health frameworks emphasize the dynamic interaction between individual characteristics, behavioral practices, and environmental conditions in shaping safety outcomes [15, 16]. Guided by these perspectives, the present study examines how perceived stress (individual factor), adherence to safety practices (behavioral factor), clinical training conditions, and equipment availability (environmental factors) jointly associated with NSI risk among nursing students.

International evidence continues to show that the problem remains widespread and multifactorial. Research from Korea, China, India, Iran, and Southern Ethiopia reveals the widespread nature of NSIs and reports several contributing factors [17–22]. According to research done in Korea, incorrect disposal of sharp instruments is one of the major causes for NSIs [18]. In India, a large number of nursing students and healthcare personnel have indicated having experienced NSIs; such behavior is often an outcome of poor preventive strategies being in place and low levels of awareness of NSIs within this population [4]. In Iran, personality and emotional intelligence, which refers to the ability to recognize and manage one’s own emotions and the emotions of others, have been linked to increased NSI risk for nurses [19].

Within this global context, evidence from the Middle East further highlights region‐specific challenges related to NSIs. NSI incidents continue to be a major occupational risk factor for healthcare workers in the Middle East and Asia. In Saudi Arabia, there have been studies showing both a high incidence of NSIs and significant gaps in knowledge, safety, and reporting [23, 24]. In Qatar, there was a retrospective study showing the majority of NSI events in operation theaters and inpatient settings, affecting nurses the most [25]. For nursing students, there was evidence from Oman showing the under‐reporting of events to be significant [26].

Despite these regional insights, the Palestinian context presents unique structural and educational challenges that warrant focused investigation. In Palestine, nursing students receive their clinical training in environments that are often overcrowded, with limited staff resources and inconsistent supervision [27, 28]. These challenges have been further intensified by the ongoing war since October 7, 2023, which has disrupted nursing education through frequent shifts to electronic distance learning amid severe infrastructural barriers and heightened psychological stress [29] and resource limitations and infrastructural constraints commonly reported in Palestinian governmental hospitals [30]. While some local studies have examined the prevalence and awareness of NSIs among nursing students, these studies have been largely descriptive in nature and have not comprehensively explored the interplay of behavioral, educational, and clinical‐environment factors. By applying multivariable analysis, this study contributes by testing whether traditional behavioral factors remain independently associated with NSI risk when psychological and environmental factors are accounted for, thereby clarifying the limitations of individual‐level prevention models in resource‐constrained settings. Consequently, there remains a need for analytically driven research that identifies factors independently associated with NSIs and examines how multiple factors collectively associated with NSI risk in this context. Therefore, this study aims (1) to determine the prevalence of NSIs among nursing students during clinical training in Palestine, (2) to examine students’ personal experiences with NSIs, including the circumstances and behaviors surrounding injury and reporting, and (3) to identify factors significantly associated with NSIs using multivariable logistic regression analysis.

## 2. Methods

### 2.1. Design and Setting

This study adopted an analytical cross‐sectional design. This study was conducted in the southern region of the West Bank. Participants were recruited from four universities that offer a Bachelor of Nursing: Palestine Polytechnic University and Hebron University in the Hebron district, and Bethlehem University and Palestine Ahliya University in the Bethlehem district. These institutions were selected because they are the only accredited universities offering a Bachelor of Nursing degree in the southern West Bank, thereby encompassing the entire accessible target population in this geographic region. Additionally, selecting these sites ensured feasible access for the research team for data collection.

### 2.2. Population and Sampling

The target population consists of nursing students enrolled in accredited Bachelor of Nursing programs in the southern West Bank, Palestine. Raosoft calculated a minimum required sample of 377 participants (95% confidence level, 5% margin of error, 50% response distribution). Eligible students were those who had completed at least one clinical training course and were willing to participate by providing informed consent. Students were excluded if they were under 20 or over 30 years of age, submitted incomplete questionnaires, or had prior professional healthcare experience, including bridging students. Excluding participants outside the specified age range ensures a homogeneous group of young nursing students at similar educational stages. Participants with prior professional healthcare experience, including bridging students, are excluded to ensure the study focuses solely on those gaining their initial clinical exposure, preserving the validity and consistency of the results. Participants were recruited using convenience sampling due to feasibility and access considerations; this approach may introduce selection bias and limit the representativeness of the sample.

### 2.3. Instrumentation

The data collection tool consisted of a structured questionnaire divided into two main sections. The first section gathered demographic and clinical training–related information, including age, gender, academic year, hepatitis B and C testing before training, perceived stress when handling needles or sharps, safety practices, and the adequacy of laboratory training on sharps and NSI prevention. This section also assessed three clinical‐environment factors using three 5‐point Likert‐scale items (1 = strongly disagree to 5 = strongly agree), namely, (a) perceived pressure from clinical staff to perform needlestick‐related procedures quickly, and (b) perceived availability of necessary equipment and supplies during clinical training. This section was developed by the research team based on previous studies [7, 23] and was further refined following feedback from faculty members, infection control specialists, and public health academics. Specifically, perceived stress when handling sharps was measured using a single, direct dichotomous item: “Do you feel stressed when handling needles or sharp instruments?” (Yes/No). This item was designed to capture the respondents’ subjective psychological discomfort or anxiety specifically in the context of sharps handling, rather than general stress.

The second section assessed students’ personal experiences with NSIs. This section began with a screening question asking whether the student had ever experienced an NSI during clinical training (Yes/No), which was used as the primary outcome variable in the analysis. Additional items asked about the number of NSIs, the procedures/activities during which they occurred, the type of sharp object involved, the clinical course during which the first NSI occurred, whether the incident was reported, and reasons for not reporting.

### 2.4. Validity and Reliability

The validity and reliability of the questionnaire were evaluated as follows. The questionnaire’s content validity was assessed through a formal review by five specialized academic research professors and infection control officers. Their comments led to some revisions to optimize the clarity and relevance of the questions. A pilot study was conducted with 25 nursing students (separate from the main study sample) to assess the questionnaire’s clarity, understandability, and ease of administration. Pilot participants reported that all items were clear and understandable. Based on their feedback, the research team reorganized the presentation of tables to improve visual layout and readability; no items were added, removed, or substantively rewritten. Pilot participants were excluded from the final sample of 398 to prevent bias and contamination of the main study data.

Regarding reliability: Most variables in this study were assessed using single, concrete items (e.g., gender, academic year, hepatitis testing status, glove use, recapping behavior, and the dichotomous yes/no measure of perceived stress). For these single‐item measures, internal consistency (Cronbach’s alpha) is not applicable, as there is only one item per construct. The clinical‐environment perception items (pressure from staff and equipment availability) were each measured with single Likert‐type items and were analyzed as separate factors rather than summed as a composite scale; therefore, calculating alpha across these three items would be inappropriate because they do not measure a single latent construct. Reliability was supported through content validity assessment, clear operational definitions, standardized administration procedures, and the pilot testing of item clarity. We acknowledge the limitation of single‐item measurement for multidimensional constructs, which is addressed in the Discussion section.

### 2.5. Data Collection Procedure

Following ethics approval, the principal investigator contacted the academic administration and clinical coordinator at the four universities to arrange for data collection and to verify eligibility. A pilot study was conducted during the preliminary data collection period from 1 to 10 October 2025. Main data collection commenced on October 15, 2025, and concluded on November 20, 2025. All 25 pilot participants were excluded from the main study sample. No changes were made to the questionnaire content following the pilot; only table formatting was adjusted.

At the beginning of every data collection session, the research team explained the study’s purpose and sampling procedures, obtained written informed consent, and handed out the paper questionnaires to the participants. Each questionnaire was assigned a numerical code to both identify the returned questionnaires and ensure participant anonymity. The researcher was always present to answer participant questions, assist to the degree necessary, and collect each questionnaire before leaving the data collection session.

To account for anticipated nonresponse and incomplete submissions, 470 questionnaires were distributed, exceeding the minimum required sample size of 377. The researcher received 415 responses (initial response rate = 93.2%). After excluding 17 incomplete or ineligible forms, the final sample comprised 398 participants, yielding an effective participation rate of 84.7% (398/470). The principal investigator collected the questionnaires each day, put them in a secure location, and has maintained confidentiality throughout the study.

### 2.6. Data Analysis

Data were analyzed using IBM SPSS Statistics version 28. Descriptive statistics (means, standard deviations, frequencies, and percentages) were used to summarize participant characteristics, clinical training–related factors, and NSI‐related experiences and to calculate the prevalence of ever having an NSI.

Bivariate associations between NSI status and categorical factors were examined using chi‐square tests, while independent‐samples *t*‐tests were used to compare mean scores of continuous variables across NSI groups. Variable selection for multivariable logistic regression was guided by both statistical screening and theoretical considerations informed by the Swiss Cheese Model and human factors framework [14–16]. All variables conceptually linked to NSI risk (academic year, stress, glove use, equipment availability) were considered for inclusion. Due to high collinearity with academic year, age was not included in the multivariable model. To avoid omitting potentially important variables due to limited bivariate power, a screening threshold of *p* ≤ 0.20 was applied, as is common in exploratory occupational health research. However, theoretically important variables (academic year, glove use, and recapping behavior) were retained regardless of their *p*‐value. This approach balances statistical rigor with theoretical grounding and is not intended as purely data‐driven variable selection.

For logistic regression, the absence of multicollinearity and the linearity of continuous factors with the logit of the outcome were assessed, with the results of these assumption checks reported alongside the model outcomes. Model fit was evaluated using the Hosmer–Lemeshow goodness‐of‐fit test. Multicollinearity was assessed using the variance inflation factor (VIF), with all values below 2.0 (range: 1.03–1.21), indicating no problematic multicollinearity among factors. Nagelkerke R^2^ was used to describe explanatory power. Adjusted odds ratios (ORs) with 95% confidence intervals (CIs) were reported, and statistical significance was set at *p* < 0.05.

### 2.7. Ethical Considerations

Ethical approval was obtained prior to conducting the study from the Ethical Committee of the Nursing College, Palestine Polytechnic University (Approval No. EA/2025/69). All data were de‐identified immediately following collection and stored securely in password‐protected electronic databases in compliance with international data protection standards equivalent to GDPR requirements. Data access was restricted to only the research team members. Before data collection, written informed consent was received from all participants, and all nurses received a detailed explanation of the study’s purpose, procedures, potential benefits, and risks, and each provided written informed consent. Participation was entirely voluntary, with the option to withdraw at any point without any negative consequences. Other issues included ensuring privacy during data collection, which we ensured by obtaining informed consent and conducting the data collection in a comfortable and private space. We assured participants that we would keep their personal information secure and confidential. The study adhered to ethical principles outlined in the Declaration of Helsinki regarding the rights, safety, and well‐being of research participants.

## 3. Results

### 3.1. Participant’s Demographics and Clinical Training–Related Factors

A total of 398 nursing students participated in the study. The mean age was 20.85 years (SD = 1.05). Most participants were female (79.9%); 52.7% were fourth‐year students, 30.7% were third‐year students, and 16.6% were in their second year. Most students (91.7%) reported undergoing hepatitis B and C testing before commencing clinical training. In terms of preclinical preparation, 81.7% indicated that laboratory training on sharps and NSI prevention was adequate.

A proportion of 39.4% of those who answered said they felt stressed when they had to use needles or sharp tools. Concerning protective practices, 78.1% always wore gloves when dealing with needles or sharps, and 72.6% consistently avoided recapping used needles. Pressure from clinical staff was noted, with 28.6% agreeing or strongly agreeing that they felt pressured to perform needlestick‐related procedures quickly. Adequate availability of necessary equipment and supplies during clinical training was reported by most students, with 40.0% agreeing and 20.1% strongly agreeing. Table 1.

**TABLE 1:** Participant`s demographics and clinical training–related factors (*n* = 398).

Variable	Categories	*N* (%)
Gender	Male	80 (20.1)
	Female	318 (79.9)

Academic year	Second year	66 (16.6)
	Third year	122 (30.7)
	Fourth year	210 (52.7)

Undergo hepatitis (B & C) testing before Training	Yes	365 (91.7)
	No	33 (8.3)

Lab training adequacy on NSI	Yes	325 (81.7)
	No	73 (18.3)

I feel stressed when handling needles or sharp instruments	Yes	157 (39.4)
	No	241 (60.6)

Do you always wear gloves when using needles or sharps?	Yes	311 (78.1)
	No	87 (21.9)

Do you always avoid recapping used needles?	Yes	289 (72.6)
	No	109 (27.4)

I feel pressured by clinical staff to perform needlestick‐related procedures quickly	Strongly disagree	35 (8.8)
	Disagree	121 (30.4)
	Neutral	128 (32.2)
	Agree	90 (22.6)
	Strongly agree	24 (6.0)

Necessary equipment and supplies were adequately available during clinical training.	Strongly disagree	18 (4.5)
	Disagree	43 (10.8)
	Neutral	98 (24.6)
	Agree	159 (40.0)
	Strongly agree	80 (20.1)

*Note: N*/*n*, number; %, percentage.

### 3.2. Prevalence and Students’ Personal Experiences With NSIs

As illustrated in Table 2, 28.6% (*n* = 114) reported experiencing at least one NSI, whereas 71.4% had never experienced such an injury. Among those who sustained an NSI, 63.2% reported only one incident, 30.7% experienced two to three incidents, and 6.1% reported more than three. The most frequently reported contexts were drawing blood (38.5%) and recapping needles (30.7%). The sharp instruments most commonly causing injuries were hollow‐bore needles (43.0%), followed by butterfly needles (35.1%). Regarding the clinical course in which the first NSI occurred, most injuries were reported during Medical and Surgical Nursing (40.4%), followed by Fundamentals of Nursing (28.9%). Most students who sustained an NSI (83.3%) reported the incident. Among those who did not, the most common reasons were lack of knowledge about the reporting procedure (78.9%), fear of blame or punishment (73.7%), perceived low risk (68.4%), and lack of time (42.1%).

**TABLE 2:** Students’ personal experiences with NSIs (*N* = 398).

Variable	Categories	*N* (%)
Have you ever experienced an NSI during your clinical training?	Yes	114 (28.6)
	No	284 (71.4)

How many times have you experienced an NSI?	Once	72 (63.2)
	2‐3 times	35 (30.7)
	> 3 times	7 (6.1)

During which activity did the injury occur? *(Select one or more)*	Administering Injections	32 (28.0)
	Drawing blood	44 (38.5)
	Disposing of sharp instruments	34 (29.8)
	Recapping needles	35 (30.7)
	Glucose check	22 (19.2)
	Others	9 (7.8)

What type of sharp object caused your most recent or most memorable injury? *(Select one or more)*	Hollow bore needle	49 (43.0)
	Butterfly needle	40 (35.1)
	Insulin syringes	26 (22.8)
	I.V cannula	30 (26.3)
	Others	15 (13.1)

In which clinical course did your first NSI occur?	Fundamental of nursing	33 (28.9)
	Medical & Surgical nursing (adult)	46 (40.4)
	Maternal and Newborn Nursing	10 (8.8)
	Pediatric Nursing	11 (9.6)
	Emergency/Critical care nursing	14 (12.3)

Did you report the NSI to a supervisor or authority ؟	Yes	95 (83.3)
	No	19 (16.7)

If No: What were the reasons for not reporting? *(Select one or more)*	Fear of blame or punishment	14 (73.7)
	Lack of Knowledge about reporting procedure	15 (78.9)
	Perceived low risk of injury	13 (68.4)
	Lack of time	8 (42.1)
	Others	6 (31.6)

*Note: N*/*n*, number; %, percentage.

### 3.3. Bivariate Associations of Explanatory Variables With NSI

Preliminary bivariate analyses were conducted to examine associations between NSI status and explanatory variables. Chi‐square tests were conducted to explore associations between categorical variables and NSI. No statistically significant association was found between academic year and NSI (*χ* ^2^ = 3.10, df = 2, *p* = 0.212); however, numerically, second‐year students had a lower NSI prevalence (19.7%) compared to third‐ and fourth‐year students (30.3% and 30.5%, respectively). Feeling stressed when handling needles or sharp instruments demonstrated a significant association with NSI (*χ* ^2^ = 10.131, df = 1, *p* = 0.001). Students who reported stress had a higher proportion of NSI (37.6%) than students who did not report stress (22.8%).

No significant associations were observed for gender, hepatitis B/C testing and vaccination, prior NSI training, wearing gloves during procedures, or avoiding needle recapping (all *p* > 0.05) (Table 3).

**TABLE 3:** Bivariate associations between categorical variables and NSI (*N* = 398).

Variables		Ever had NSI		X 2	df	*p*‐value
		Yes *n* (%)	No *n* (%)			
Gender	Male	24 (30.0)	56 (70.0)	0.090	1	0.764
	Female	90 (28.3)	228 (71.7)			

Academic year	2nd year	13 (19.7)	53 (80.3)	3.099	2	0.212
	3rd year	37 (30.3)	85 (69.7)			
	4th year	64 (30.5)	146 (69.5)			

Undergone hepatitis B&C testing and vaccine	Yes	107 (29.3)	258 (70.7)	0.972	1	0.324
	No	7 (21.2)	26 (78.8)			

Training on NSI	Yes	91 (28.0)	234 (72.0)	0.359	1	0.549
	No	23 (31.5)	50 (68.5)			

I feel stressed when handling needles or sharp instruments	Yes	59 (37.6)	98 (62.4)	10.131	1	**0.001**
	No	55 (22.8)	186 (77.2)			

Do you always wear gloves when using needles or sharps?	Yes	86 (27.7)	225 (72.3)	0.683	1	0.409
	No	28 (32.2)	59 (67.8)			

Do you always avoid recapping used needles?	Yes	82 (28.4)	207 (71.6)	0.038	1	0.846
	No	32 (29.4)	77 (70.6)			

*Note:* *χ* ^2^: Pearson chi‐square test statistic. *n* = number; % = percentage. Bold indicates significant < 0.05.

Abbreviation: df, Degrees of freedom.

Independent‐samples *t*‐tests were used to compare the mean age, pressure from clinical staff, and equipment availability across NSI groups. Age, perceived availability of necessary equipment, workload, and pressure by clinical staff did not significantly differ between groups (Table 4).

**TABLE 4:** Comparison of mean age, pressure from clinical staff, and equipment availability across NSI groups (*N* = 398).

Variable	Ever had NSI	*N*	Mean	SD	*t*‐test	*p*‐value
Age	Yes	114	20.98	0.93	−1.53	0.126
	No	284	20.82	1.071		

I feel pressured by clinical staff to perform needlestick‐related procedures quickly	Yes	114	2.88	1.07	−0.124	0.901
	No	284	2.86	1.04		

Necessary equipment and supplies were adequately available during clinical training.	Yes	114	3.74	1.00	−1.59	0.112
	No	284	3.55	1.08		

*Note: N*/*n* = number. *t*: *t*‐test statistic.

Abbreviation: SD, standard deviation.

### 3.4. Multivariable Logistic Regression Analysis

Logistic regression was performed to identify factors associated with NSI among nursing students (*n* = 398). The overall model was statistically significant compared with the null model, *χ* ^2^(6, *n* = 398) = 17.05, *p* = 0.009, and explained about 6% of the variance in ever having an NSI (Nagelkerke R^2^ = 0.060). The Hosmer–Lemeshow test indicated acceptable calibration, *χ* ^2^(8) = 7.25, *p* = 0.510.

After adjustment for the other variables, students who reported feeling stressed when handling needles or sharp instruments had significantly higher odds of experiencing an NSI compared with those who did not feel stressed (aOR = 2.13; 95% CI: 1.35–3.37, *p* = 0.001). Academic year, glove use, avoiding recapping, and perceived equipment availability were not significantly associated with NSI in the multivariable model (all *p* > 0.05). Although academic year was not statistically significant in bivariate analysis (*p* = 0.212), it was retained in the model due to its theoretical relevance; it remained nonsignificant in the adjusted analysis. Given the low explanatory power of the model (Nagelkerke *R* ^2^ = 0.060), these findings should be interpreted as identifying statistical associations rather than providing a comprehensive predictive model of NSI risk. Furthermore, due to the cross‐sectional design, the direction of the association with stress cannot be established; it is plausible that experiencing an NSI increases subsequent perceived stress when handling sharps, rather than stress preceding and causing the injury (see Table 5).

**TABLE 5:** Multivariable logistic regression analysis.

Factors	B	SE	Wald	*p*‐value	OR	95% CI for OR
Academic year (Ref: 4th year)	—	—	2.43	0.297	—	—
2nd year vs 4th year	−0.48	0.35	1.81	0.179	0.62	0.31–1.24
3rd year vs 4th year	0.10	0.26	0.14	0.709	1.10	0.67–1.83
Stress when handling needles/sharps (Ref: No)	0.76	0.23	10.55	0.001	2.13	1.35–3.37
Always wears gloves (Ref: Does not always wear)	−0.29	0.28	1.07	0.301	0.75	0.43–1.30
Always avoid recapping (Ref: Does not always avoid)	0.16	0.26	0.35	0.554	1.17	0.70–1.96
Equipment availability (Likert 1–5)	0.20	0.11	3.17	0.075	1.22	0.98–1.53
Constant	−1.82	0.52	12.32	< 0.001	0.16	—

*Note:* B = unstandardized regression coefficient. Wald = Wald chi‐square statistic. Ref = reference category. SE = standard error of B.

Abbreviations: CI, confidence interval; OR, odds ratio.

To address potential confounding, we conducted a sensitivity analysis using a forced‐entry logistic regression model that included gender as an a priori confounder—selected due to its established association with both perceived stress and injury risk in the broader occupational health literature—along with the variables from the primary model. As shown in Supporting Table 1, the results were materially unchanged: perceived stress remained the sole significant independent factor associated with NSI (aOR = 2.15; 95% CI: 1.36–3.41, *p* = 0.001). Gender was not significantly associated with NSI in the adjusted model (*p* = 0.615). This sensitivity analysis supports the robustness of the primary findings.

## 4. Discussion

The findings show that the prevalence rate of at least one NSI was 28.6% among nursing students, with blood sampling and recapping of needles topping the injury incidents and hollow‐bore needles ranking highest among devices involved in injuries. Fear and lack of knowledge remained barriers to reporting. It can therefore be hypothesized that safety at nursing training facilities in Palestine continues to face numerous challenges related to overcrowded training environments and limited availability of resources and safety devices. However, these structural factors (overcrowding, resource limitations, supervision gaps) were not directly measured in this study. The following discussion of these factors is offered as potential explanations based on the existing Palestinian literature, not as conclusions directly supported by our data. Future research should measure these structural variables directly.

The demographic distribution in this study reflects the typical composition of nursing programs in Palestine, where the majority of participating students were females; this aligns with previous findings [23, 31, 32]. Stress was identified by 39.4% of participants and can be especially significant within the Palestinian environment because students find themselves practicing in high‐stress wards that lack equipment and supervisory guidance while still being subjected to psychosocial stress factors in a politically unstable environment [33–35]. This is consistent with evidence of substantial psychological burden and burnout experienced by nurses working in similar conflict‐affected and resource‐limited settings [33, 36].

Although there were extensive preparation efforts, 91.7% of students underwent hepatitis B/C screening, and 81.7% found lab training adequate; however, it was found that there still exist major challenges within the hospital setting. Moreover, 28.6% agreed or strongly agreed with feeling pressured by hospital staff to work at a fast pace; indeed, 39.9% found equipment and facilities to be inadequate, reflecting systemic constraints typical of resource‐limited Palestinian hospitals. These findings closely mirror regional evidence documenting gaps in infection‐control adherence arising from equipment shortages, limited training opportunities, and inconsistent implementation of infection‐control policies [27, 28, 37, 38].

The current prevalence of 28.6% is a new high when compared to the previous Palestinian nursing student statistic of 23.4% [7], but it is still below the global pooled estimate of 35.7% reported recently [22] and those published in Italy, 39.0% [39], and India, 30.3% [4]. The considerable increase in Palestinian governmental hospital overcrowding and lack of staffing may contribute to the recognition of this higher prevalence and its implication for student safety during clinical training. A blood draw and needle recapping, which is the practice of putting the cap back on a used needle, made up the majority of activities resulting in NSIs in this study. The practice of recapping needles is globally prohibited. However, the ongoing issue is a result of several barriers that have been well documented in the Palestinian clinical environment, including long‐distance travel or overflowing sharps containers and fears associated with being reprimanded when leaving a used needle uncovered. Hollow‐bore needles were responsible for 43% of injuries, aligning with the other local literature [7]. Because Palestinian hospitals do not have access to a wide variety of safety‐engineered devices, they continue to use a large number of hollow‐bore devices, creating a higher likelihood of injury than hospitals in other countries.

Despite the heavy burden of NSIs in Palestine, reporting behavior is generally positive (83.3%) compared with much lower rates from studies conducted in Saudi Arabia, China, and the Philippines [23, 40, 41]. This likely indicates the positive impact of increased needlestick awareness campaigns at some Palestinian universities. However, fear of blame and lack of procedural knowledge continue to be major barriers reported by study participants—though it is important to note that these reasons were provided by a small subset of only 19 students who did not report, and thus must be interpreted with caution—as has also been reported in studies from other countries in the Middle East [23]. These findings should be interpreted with caution, as self‐reported data may be subject to recall bias and social desirability bias, potentially leading to underreporting of unsafe practices or over‐reporting of compliance with safety measures.

The results of the bivariate analysis confirmed a significant association between perceived stress and the occurrence of NSIs, while academic year was not significantly associated. In the adjusted analysis, stress remained the sole significant independent factor. However, the cross‐sectional nature of this study limits the ability to infer directionality. While it is plausible that stress impairs concentration and increases NSI risk, it is equally plausible that experiencing a prior NSI leads to heightened anxiety and perceived stress when subsequently handling sharps [42]. Therefore, this association must be interpreted as correlational rather than causal. Furthermore, the low explanatory power of the model indicates that stress alone accounts for a small fraction of NSI risk, and most of the variability in NSI occurrence remains unexplained by our model. Nevertheless, this finding aligns with regional evidence showing that emotional stress imposes significant challenges to safely handling needles [19] and that resource scarcity, inconsistent supervision, and large sociopolitical stressors in Palestine exacerbate the issue [33]. Consistent with a numerical trend in our data, senior students often report higher injury rates. This may be because senior students are frequently placed in acute departments characterized by excessive caseloads and insufficient staffing, often assuming responsibilities that would typically be assigned to junior nurses [43]. However, the lack of statistical significance for academic year in our bivariate analysis suggests that these differences may be confounded by other factors, such as stress or clinical workload.

This study found no significant association between glove use or recapping and NSIs. Consistent with Kim and Moon [18] and Basaza et al. [44], safety behaviors did not reliably predict injuries, indicating that contextual factors may be more influential. However, this apparent contradiction may be explained by contextual factors such as high workload, limited supervision, and resource constraints, which may reduce the effectiveness of individual safety practices. This may also reflect a gap between knowledge or reported behaviors and actual practice in complex clinical environments. The model demonstrated low explanatory power, suggesting that additional unmeasured structural and environmental factors may contribute to NSI risk.

### 4.1. Limitations

This study has several limitations. The cross‐sectional design precludes causal inference or temporal sequencing between factors and NSI. Consequently, all reported associations are correlational, and identifying a variable as independently associated with the outcome does not imply causality. Convenience sampling and recruitment from the southern West Bank limit external generalizability to all Palestinian nursing students.

Self‐report data introduce recall and social desirability bias. Students may misremember injury circumstances or underreport unsafe behaviors (e.g., recapping needles) to present themselves favorably. Although our reporting rate (83.3%) is higher than many published studies, suggesting relatively open reporting, these biases may still distort findings. Future studies should consider anonymous electronic reporting or direct observation.

Single‐item measures were used for perceived stress, equipment availability, and pressure from clinical staff. While appropriate for concrete, unidimensional constructs and to reduce respondent burden, they may not capture full multidimensional complexity. Multi‐item validated scales (e.g., Perceived Stress Scale, NASA‐TLX) would provide greater psychometric robustness. Additionally, test–retest reliability was not calculated for these single items. Excluding students with prior clinical experience or outside the 20–30 age range enhances internal homogeneity but reduces generalizability to older or bridging students.

Structural factors (e.g., clinician supervision numbers, safety‐engineered device availability, institutional safety climate, patient‐to‐student ratios) were not measured. The study relied on subjective perceptions of workload and pressure, which may not fully reflect actual clinical conditions. The logistic regression model explained only 6% of NSI variance (Nagelkerke R^2^ = 0.060), indicating that measured variables account for a small fraction of NSI risk. Unmeasured factors likely include safety climate, device availability, supervision ratios, and training quality. The modest number of NSI events (*n* = 114) limited statistical power to detect smaller effects or interaction terms. Nonsignificant findings for glove use, academic year, and equipment availability may reflect Type II error (false negatives).

## 5. Recommendations and Implications

### 5.1. Nursing Education

◦ Integrate mandatory stress‐management and resilience training into the curriculum, directly addressing the study’s finding that perceived stress when handling sharps is significantly associated with NSI occurrence.
◦ Introduce structured competency checklists and supervised procedure sign‐offs before independent practice, specifically targeting high‐risk procedures identified in this study, such as blood drawing and needle recapping.

### 5.2. Clinical Training Sites and Hospitals

◦ Enforce minimum nurse‐to‐student supervision ratios for invasive procedures. This recommendation is justified by local literature indicating that Palestinian training environments are often overcrowded with limited staff resources [27, 28], which aligns with our finding that 28.6% of students felt pressured by clinical staff to perform procedures quickly.
◦ Prioritize the procurement of safety‐engineered devices and ensure the availability of strategically placed sharps containers. This is directly supported by our finding that hollow‐bore needles caused 43% of injuries, combined with local evidence noting the lack of safety‐engineered devices in Palestinian hospitals [7].
◦ Establish blame‐free reporting systems with simplified, student‐friendly post‐exposure protocols, directly addressing the barriers identified in this study where fear of blame (73.7%) and lack of knowledge about reporting procedures (78.9%) were the primary reasons for nonreporting among the unreported cases.

### 5.3. Policy & Institutional Level

◦ Implement targeted awareness campaigns to destigmatize NSI reporting and educate students on reporting procedures, directly responding to the knowledge gaps identified in this sample.
◦ Include NSI prevention and reporting indicators in hospital accreditation and university program evaluations to ensure adherence to safety protocols, addressing the systemic constraints highlighted in the regional literature [27, 28, 35, 36].

## 6. Conclusion

This study found that NSIs remain relatively common among nursing students in Palestine, with a prevalence of 28.6%. The findings suggest that perceived stress when handling sharps is significantly associated with NSI occurrence, while other examined factors were not statistically significant in the adjusted analysis. Given the cross‐sectional design, these associations should not be interpreted as causal. The relatively low explanatory power of the model indicates that additional unmeasured factors may contribute to NSI risk.

These findings highlight the importance of addressing stress and reinforcing safe clinical training environments. Interventions such as enhanced supervision, stress management strategies, and supportive reporting systems may be beneficial; however, further longitudinal and interventional research is needed to better understand causal pathways and inform effective prevention strategies.

## Author Contributions

• Fuad Farajalla: conceptualization; methodology; formal analysis; writing–original draft; writing–review and editing.

• Nesreen Alqaissi: conceptualization; supervision; validation; writing–review and editing.

• Yousef M. Jaradat: conceptualization; writing–review and editing.

• Mohammad Qtait: conceptualization; validation; writing–review and editing.

• Areej Ashraf: investigation; writing–review and editing.

• Tasneem Ghannam: investigation; writing–review and editing.

• Bagdad Ajwah: investigation; writing–review and editing.

• Amal Najada: investigation; writing–review and editing.

• Sujood Dawadeh: investigation; writing–review and editing.

## Funding

The authors received no financial support for the research, authorship, and/or publication of this article.

## Disclosure

All authors read and approved the final version of the manuscript.

## Conflicts of Interest

The authors declare no conflicts of interest.

## Supporting Information

Additional supporting information can be found online in the Supporting Information section.

## Supporting information


**Supporting Information** The following supporting files are available with this article: STROBE Checklist: A completed Strengthening the Reporting of Observational Studies in Epidemiology (STROBE) checklist to ensure transparent reporting of the quantitative component. Supporting Table 1: Results of the Forced‐Entry Sensitivity Logistic Regression Analysis Including Gender as an A Priori Confounder (*n* = 398).

## Data Availability

Data are available from the corresponding author upon reasonable request.
