# Supplementary material for: Needlestick and Sharps Injuries Among Nursing Students in Palestine: Prevalence, Reporting Practices, and Associated Factors
Source: Nurs Res Pract. 2026 Sep 15;2026:4472112. doi: 10.1155/nrp/4472112 (PMC13575877; doi:10.1155/nrp/4472112)
Supplement: Supplementary file 1 [file NRP-2026-4472112-s001.zip › STROBE.docx]

***Study Title:***

**Needlestick and Sharps Injuries among Nursing Students in Palestine: Prevalence, Reporting Practices, and Associated Factors**

| **STROBE Item** | **Recommendation** | **Where Reported** |
| --- | --- | --- |
| **Title & Abstract** |  |  |
| 1(a) | Indicate study design in title or abstract | Abstract (“A cross-sectional study…”) |
| 1(b) | Provide an informative summary of methods and findings | Abstract |
| **Introduction** |  |  |
| 2 | Describe scientific background and rationale | Introduction, pp. 1–3 |
| 3 | State study objectives and hypotheses | End of Introduction |
| **Methods** |  |  |
| 4 | Present key elements of study design | Methods – Design & Setting |
| 5 | Describe setting, locations, and dates | Methods – Setting & Data Collection |
| 6(a) | Detail eligibility criteria and sampling | Methods – Population & Sampling |
| 7 | Define outcomes, exposures, predictors | Methods – Instrumentation |
| 8 | Data sources and measurement methods | Methods – Instrumentation |
| 9 | Describe efforts to address bias | Validity & Reliability section |
| 10 | Explain sample size calculation | Methods – Population & Sampling |
| 11 | Handling of quantitative variables | Methods – Data Analysis |
| 12(a) | Describe statistical methods | Methods – Data Analysis |
| 12(b) | Subgroup or interaction analyses | Not applicable |
| 12(c) | Handling of missing data | Described indirectly (“17 incomplete… excluded”) |
| 12(d) | Consideration of sampling strategy | Convenience sampling limitations acknowledged |
| 12(e) | Sensitivity analyses | Not applicable |
| **Results** |  |  |
| 13(a) | Report number of participants at each stage | Results – Data Collection & Sample |
| 13(b) | Provide reasons for non-participation | Excluded incomplete or ineligible forms (n=17) |
| 13(c) | Flow diagram | Not included (optional) |
| 14(a) | Participant characteristics | Table 1 |
| 14(b) | Missing data for each variable | Not explicitly stated (minimal) |
| 15 | Outcome measures | Tables 2–5 |
| 16 | Estimates, precision, and confounders | Table 5 |
| 17 | Additional analyses | Bivariate + logistic regression |
| **Discussion** |  |  |
| 18 | Summary of key findings | First paragraphs of Discussion |
| 19 | Study limitations | Limitations section |
| 20 | Interpretation considering context & evidence | Discussion section |
| 21 | Generalizability | Limitations (external validity discussed) |
| **Other Information** |  |  |
| 22 | Funding and role of funders | Declared as none (“Funding” section) |
