# Supplementary material for: Needlestick and Sharps Injuries Among Nursing Students in Palestine: Prevalence, Reporting Practices, and Associated Factors
Source: Nurs Res Pract. 2026 Sep 15;2026:4472112. doi: 10.1155/nrp/4472112 (PMC13575877; doi:10.1155/nrp/4472112)
Supplement: Supplementary file 1 [file NRP-2026-4472112-s001.zip › Supplementart Table 1.docx]

Supplementary Table 1. Forced-Entry Sensitivity Logistic Regression Analysis for Factors Associated with NSI, including Gender as an A Priori Confounder (N = 398)

| Predictor | B | SE | Wald | p-value | OR | 95% CI for OR |
| --- | --- | --- | --- | --- | --- | --- |
| Academic year (Ref: 4th year) | – | – | 2.40 | .302 | – | – |
| 2nd year vs 4th year | -0.48 | 0.35 | 1.83 | .176 | 0.62 | 0.31 – 1.24 |
| 3rd year vs 4th year | 0.09 | 0.26 | 0.11 | .740 | 1.09 | 0.66 – 1.81 |
| Stress when handling needles/sharps (Ref: No) | 0.77 | 0.23 | 10.74 | .001 | 2.15 | 1.36 – 3.41 |
| Always wears gloves (Ref: Does not always wear) | -0.28 | 0.28 | 0.95 | .330 | 0.76 | 0.44 – 1.32 |
| Always avoids recapping (Ref: Does not always avoid) | 0.18 | 0.27 | 0.45 | .504 | 1.20 | 0.71 – 2.02 |
| Equipment availability (Likert 1–5) | 0.20 | 0.11 | 3.20 | .074 | 1.22 | 0.98 – 1.53 |
| Gender (Ref: Female) | -0.15 | 0.29 | 0.25 | .615 | 0.86 | 0.49 – 1.52 |
| Constant | -1.59 | 0.69 | 5.31 | .021 | 0.20 | — |

*Note. B = Unstandardized regression coefficient; SE = Standard error; Wald = Wald chi-square statistic; OR = Odds ratio; CI = Confidence interval. Gender was forced into the model as an a priori confounder based on established literature linking gender to both perceived stress and injury risk.*
